# MicroRNA miR-126 attenuates brain injury in septic rats via NF-κB signaling pathway

**DOI:** 10.1080/21655979.2021.1937905

**Published:** 2021-06-11

**Authors:** Anna Nong, Qingfeng Li, Zhijing Huang, Yunan Xu, Kebin He, Yuying Jia, Zhenyi Cen, Lianghua Liao, Yueyan Huang

**Affiliations:** a Graduate School, Youjiang Medical University for Nationalities, Baise, Guangxi China; b Department of Radiology, Affiliated Hospital of Youjiang Medical University for Nationalities, Baise, Guangxi China; c Department of Pediatric Internal Medicine Ward 1, Affiliated Hospital of Youjiang Medical University for Nationalities, Baise, Guangxi China

**Keywords:** Sepsis, blood-brain barrier, brain injury, miR-126, nf-κB signaling pathway

## Abstract

The purpose of this study was to investigate the impact and mechanism of microRNA miR-126 on brain injury induced by blood-brain barrier (BBB) damage in septic rats. We used cecal ligation and perforation (CLP) to create a rat model of sepsis. The experimental rats were randomly divided into Control group, CLP group, CLP + miR-NC group, CLP + miR-126 group and CLP + miR-126 + NF-κB pathway agonist (PMA) group. MiR-126 expressed in the brain tissue of CLP rats was down-regulated by qRT-PCR. Upregulation of miR-126 in CLP rats could improve brain injury and BBB marker protein level, reduce brain water content, Evans blue extravasation, inflammation, and excessive oxidative stress. This could also result in an inhibition of NF-κB signaling pathway activity. In conclusion, miR-126 overexpression can prevent brain injury caused by BBB damage via the inhibition of NF-κB signaling pathway activity.

## Graphical Abstract

**Figure:**
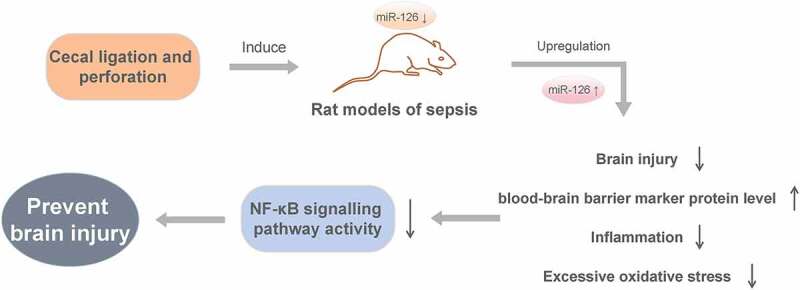

## Introduction

Urinary, respiratory, digestive tract, and central nervous system infections or trauma, surgery and other factors could result in a systemic inflammatory response syndrome (SIRS) known as sepsis [1]. Sepsis can easily lead to multiple organ dysfunction and is associated with a high mortality [2]. This disease has been demonstrated to threaten the life safety of patients. Incidence and mortality rates related to sepsis remain high even with the progress made in the field of diagnosis and treatment. Patients with sepsis-associated encephalopathy (SAE) account for about 52% of the total number of sepsis patients; the more severe the encephalopathy is, the higher the mortality rate is [3]. SAE patients often suffer from serious cognitive dysfunction, resulting in a decline in the quality of life. SAE is reported to be a consequence of neurotoxic substances entering the brain due to changes in blood-brain barrier (BBB) permeability, inducing insufficient cerebral perfusion and neuroinflammation [4]. Other studies have pointed out that disturbance on the body’s inflammatory response balance by sepsis is the key to multiple organ failure [5]. However, the pathological changes of SAE are very complex, and there is not yet a good treatment plan. Therefore, further analysis and research are needed.

Non-coding short-chain RNA with a length of around 20 nt is known as microRNA (miRNA). Research has demonstrated that miRNAs are key in the manifestation of diseases [6]. In terms of sepsis, many miRNAs are regarded as biomarkers or prognostic indicators for patients [7], suggesting that miRNAs are closely related to sepsis. Studies have shown that overexpressed miR-210 and miR-494 are key in inducing acute kidney injury (AKI) in septic individuals and they have the potential to be the prognosis indicators of AKI [8]. Tong et al. [9] found that upregulation of miR-146b-5p suppressed KLF4 expression and was involved in intestinal injury in sepsis. Song et al. [10] showed that downregulation of miR-122-5p inhibited lipopolysaccharide-induced oxidative stress, inflammation and apoptosis in cardiomyocytes, thereby preventing sepsis-induced myocardial injury. These studies have shown that miRNAs play a crucial role in sepsis-induced organ failure. On the other hand, Chen et al. discovered a significant decrease in serum obtained from septic patients; the degree of miR-126-3p downregulation is in correlation with septic severity [11]. Meanwhile, research has revealed that upregulation of miR-126 expression in septic rats is able to activate AKT/Rac1 signaling pathway, thereby inhibiting sepsis inflammation and improving the prognosis of septic mice [12]. However, there are few studies on the miR-126 involvement in SAE progression.

Nuclear factor kappa B (NF-κB) is a broad range of transcription factors that control a variety of biological processes, including inflammation and apoptosis [13]. Shang et al. [14] showed that butorphanol could have a therapeutic effect on brain injury in septic rats by inhibiting the NF-κB signaling pathway. Funahashi et al. [15] found that miR-146a inhibited NF-κB signaling pathway activation and prevented sepsis-induced multi-organ damage. However, studies on miR-126 regulating NF-κB signaling pathway in sepsis have not been reported. Therefore, we speculated that miR-126 could also ameliorate sepsis-induced brain injury by inhibiting the activity of NF-κB signaling pathway. This study explores the effects of miR-126 on SAE and their mechanism by constructing cecal ligation and perforation (CLP) rat model of sepsis, in order to establish a theoretical basis to revolve around therapeutics of SAE.

## 1 Material and methods

### 1.1 Establishment and grouping of a sepsis rat model

Thirty-six male SD rats aged 6 to 8 weeks, weight 180–220 g, were chosen and randomized into six groups. After one week of adaptive feeding, septic rat models were established by CLP. Preoperative fasting for 24 h was conducted with the exception of unlimited water drinking. Post-fasting subjects were injected with anesthesic intraperitoneally containing 10% chloral hydrate (0.3 g/kg); no peritonitis, pain, discomfort, and other symptoms were observed after injection. Anesthesia depth was determined by the pinch test. Laparotomy was used to expose the cecum post-general-anesthesia. Ligation of the cecum was done at the 1/2 mark. In the area with the least blood vessels, a needle was used to puncture the cecum followed by the removal of a minimal quantity of content from the pierced site. After this, the cecum was relocated and the wound sutured. Subcutaneous injection of 30 mL/kg saline for anti-shock was used to finish creating the CLP model. In the control group, the abdominal cavity was closed without any operation after laparotomy. Six hours after CLP model operation, experimental groups were injected via the tail vein with 10 μg negative agomir (CLP + miR-NC group), 10 μg miR-126 agomir (CLP + miR-126 group), 2 μg/kg NF-κB pathway agonist (PMA) (CLP + PMA group) [16], and miR-126 agomir combined with PMA (CLP + miR-126 + PMA group). Twenty-four hours after CLP modeling, rats in different treatment groups were examined for various parameters, followed by venous blood taken from rats’ orbital vein; then rats were euthanized to collect rat brain tissue. MiR-126 agomir was obtained from Guangzhou RiboBio Co., LTD (Guangzhou, China). The concentration of miR-126 agomir was 10 μg per rat every 3 days as previously described [17]. This study was approved by the Medical Ethics Committee of Affiliated Hospital of Youjiang Medical University for Nationalities (2020–12-8).

## 1.2 qRT-PCR

Brain tissue from rats in each treatment group (n = 3 per group) was used to extract total RNA using the TRizol method. A random primer reverse transcription kit (Thermo, USA) was used to prepare the cDNA after RNA concentration and purity were measured. The SYBR GREEN kit (TaKaRa, Japan) was used following protocols to distinguish the expression level of miR-126. U6 was used as the internal reference with the addition of six replicas arranged for the study. Quantitative analysis with the 2 ^− ΔΔCt^ method was used to calculate the relative expression of target gene.

### 1.3 Neurobehavioral assessment

Three experimenters were blinded to the neurobehavioral assessment of rats (n = 6 per group). After 24 h of CLP modeling, rats were evaluated for auricular, corneal, righting, tail-flick, and escape reflexes. The criteria for assessing reflexes are as follows: 0 = absent, 1 = diminished (within 10 min), and 2 = normal. The most that could be scored was 10 points; the lower the score, the more serious the brain injury was.

### 1.4 Detection of brain water content

The dry-wet ratio method was used to determine the water content present in rat brain tissue within the individual groups (n = 3 per group). The collected brain tissue was removed from the olfactory bulb, cerebellum, and low brainstem and was weighed at once, and the value was considered as the wet weight. The dry weight of the brain tissue was measured after drying of the tissue sample (85°C, 24 h) and the contents of water were calculated as previously described [18].

### 1.5 Evans blue (EB) extravasation method to detect BBB permeability

After 24 h of CLP modeling, rats (n = 3 per group) were intravenously injected with 45 mg/kg EB dye (Sigma-Aldrich Corp). After 2 h, 18 mg/kg pentobarbital sodium was injected intraperitoneally. After completed anesthesia, the thoracic cavity was opened and perfused with heparin saline (0.9% sodium chloride + 20 U/mL heparin sodium) into the left ventricle. Rats were executed 2 min later and the brain tissue was immediately isolated and stored overnight in a refrigerator at 4°C to measure the wet weight. The brain tissue was incubated with 150 μL of formamide at 70°C for 8 h, then centrifuged at 4°C for 5 minutes at 6000 r/min before an enzyme-labeled instrument was used to determine the absorbance of the supernatant at 620 nm. Based on the standard curve of EB, the concentration of EB in brain tissue and plasma was calculated. BBB permeability was presented by the content of EB, which is equal to the concentration of EB over the brain wet weight (μg/g).

### 1.6 ELISA detection

#### 1.6.1 Detection of inflammatory factors in serum

The venous blood (n = 6 per group) was put under 4°C and centrifuged at 1000 r/min for 10 minutes. The supernatant of the serum was moved into a new centrifuge tube, packed, and stored at −80°C. ELISA kit was used to detect TNF-α, IL-6, IL-1β, and IL-10 levels in serum according to kit instructions.

#### 1.6.2 Detection of oxidative stress-related indicators in brain tissue

The homogenizer tube was used to homogenize 30 mg of isolated rat brain tissue (n = 3 per group). Homogenized tissue was then centrifuged at 4°C at 9000 r/min for 10 min before the supernatant was collected in the new centrifuge tube. Brain tissue catalase (CAT), superoxide dismutase (SOD), and malondialdehyde (MDA) activities were acquired by the ELISA kit.

### 1.7 Western blot

A nuclear protein extraction kit (Sigma-Aldrich, USA) was used to obtain NF-κB (p65) and p65 in the nucleus and cytoplasm, respectively, from rat brain tissue in each group (n = 3 per group). RIPA buffer was used in the extraction of protein. A BCA kit (Thermo, USA) was used to calculate the concentration of protein before it underwent separation using a 10% SDS-PAGE gel. The product was then transported to the PVDF membrane. The sample was then placed in 5% skim milk for 1 hour for blocking. This was then incubated with the primary antibody at 4°C overnight. The sample was further incubated at room temperature with the relative secondary antibody for one hour the day after. After washing with TBST, ECL chemiluminescence substrate was added and reacted for 3 min. The gel imaging system was used to collect images with the Image LabTM Software incorporated to examine the gray bands of the protein bands gained. Cytoplasmic proteins were normalized using β-actin as an internal reference, while nuclear proteins were normalized using H3 as an internal reference.

### 1.8 Statistical analysis

SPSS 25.0 was used for statistical analyses. Data were presented as mean ± standard deviation (SD). For comparison, one-way ANOVA followed by LSD t-test or Student’s t-test was used to evaluate difference between more than two groups or two groups, respectively. P < 0.05 was considered as significant difference.

## 2 Results

### 2.1 Downregulation of miR-126 expressed in septic rat brain tissue

**Figure 1.**
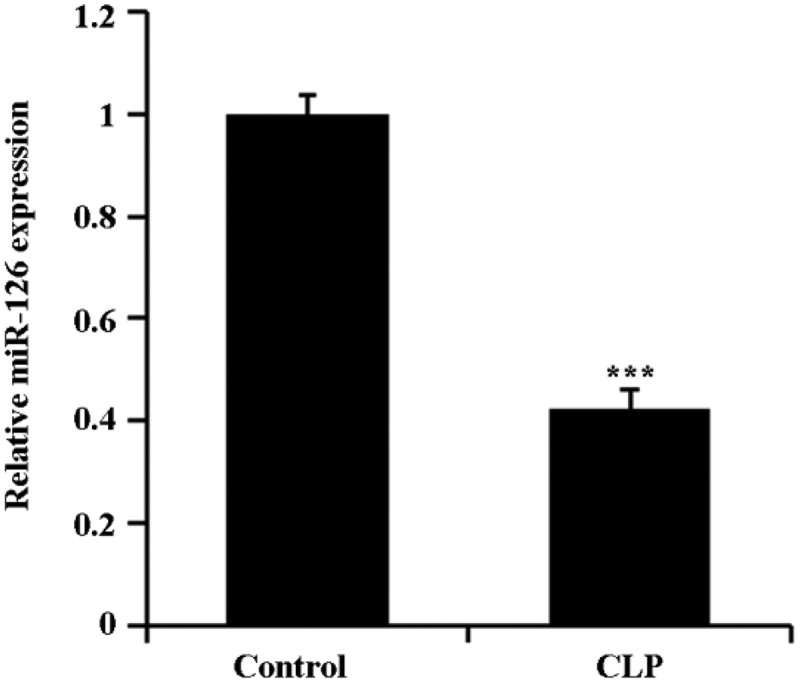
Down-regulation of miR-126 expression in brain tissue of CLP rats

The expression of miR-126 in brain tissues of CLP rats was first detected by qRT-PCR. The results showed that the level of expressed miR-126 in the brain tissue of CLP rats was notably decreased in comparison with the control group (Figure 1, P < 0.05).

Brain tissues were collected from each group of rats, and the expression of miR-126 in the tissues was detected by qRT-PCR. ***P < 0.001.

## 2.2 miR-126 can improve the neurobehavioral score of septic rats

**Figure 2.**
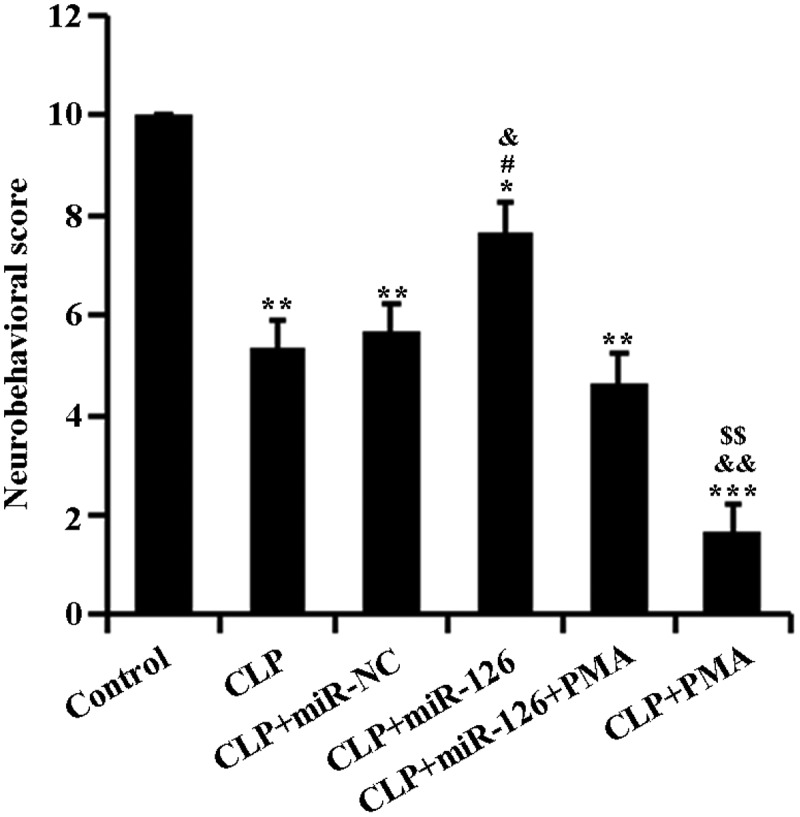
Neurobehavioral scores of rats in different treatment groups

To determine the effect of miR-126 on brain injury in septic rats, we performed neurobehavioral scoring on each group of rats. The control group was scored 10 points in the neurobehavioral evaluation. In comparison, scores of rats in the experimental groups dropped remarkably: in comparison to the CLP + miR-NC group, there was a massive rise in the scores of CLP + miR-126 group; compared to CLP + miR-126 + PMA group, CLP + miR-126 group’s score remarkedly increased and that of CLP + PMA group decreased. When compared to CLP group, CLP + PMA group had scores that conspicuously decreased (Figure 2, P < 0.05). Therefore, it can be concluded that miR-126 may enhance neurobehavioral activity in septic rats. It can also weaken the inhibitory effect of PMA on the neurobehavioral activities of septic rats.

The rats in each group were neurobehaviorally assessed after 24 h of treatment. *P < 0.05 vs. control group, ** P < 0.01 and ***P < 0.001; ^#^P < 0.05 vs. CLP + miR-NC group; ^&^P < 0.05 vs. CLP + miR-126 + PMA group, ^&&^P < 0.01; ^$^P < 0.05 vs. CLP group.

### 2.3 Effect of miR-126 on BBB in septic rats

**Figure 3.**
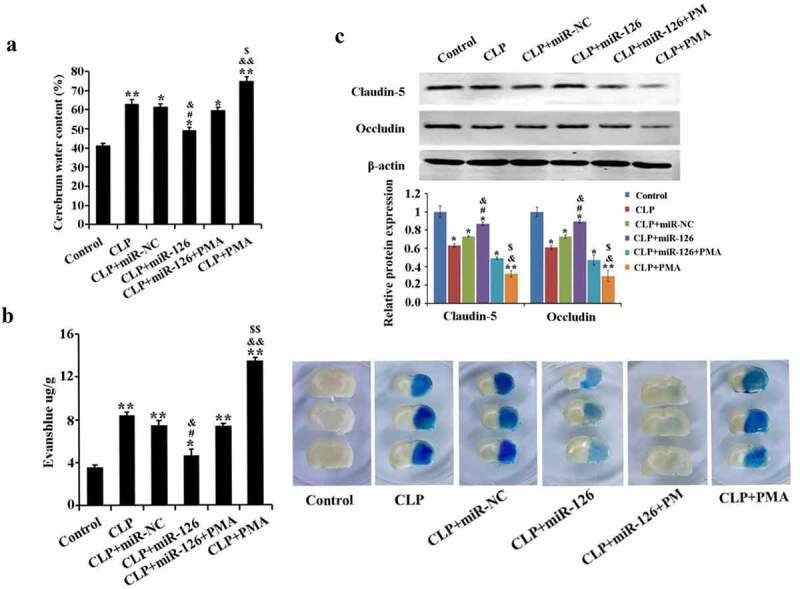
Water content and EB extravasation in brain tissue of rats in different treatment groups

The contents of water and EB extravasation in rat brain tissue from CLP group remarkably increased with a significant drop in expression levels of BBB-related proteins claudin-5 and occludin, in comparison with the control group. In contrast, after overexpression of miR-126, the brain tissue water content and EB extravasation in CLP-induced rats were significantly reduced, and the expression of claudin-5 and occludin was significantly increased. In addition, the mitigating effect of miR-126 on brain injury was eliminated in the CLP+miR-126 group when overexpression of miR-126 was accompanied by activation of NF-κB signaling pathway (Figure 3a-C, P < 0.05). In conclusion, miR-126 could reduce the water content of brain tissue in septic rats and BBB permeability, promote BBB-related protein expression, and alleviate the damage of BBB in septic rats. It can weaken the effect of PMA on BBB.

A. Determination of water content in brain tissue of rats in each group by dry-wet specific gravity method; B. Evans blue extravasation method to detect blood-brain barrier permeability C. Western blot to detect the expression of claudin-5 and occludin proteins in rats of each group. *P < 0.05, **P < 0.01 vs. control group; ^#^P < 0.05 vs. CLP + miR-NC group; ^&^P < 0.05, ^&&^P < 0.01 vs. CLP + miR-126 + PMA group; ^$^P < 0.05, ^$$^ P < 0.01 vs. CLP group.

**2.4 Effects of miR-126 on serum levels of inflammatory cytokines TNF-α, IL-6, IL-1β, and IL-10 in septic rats**

**Figure 4.**
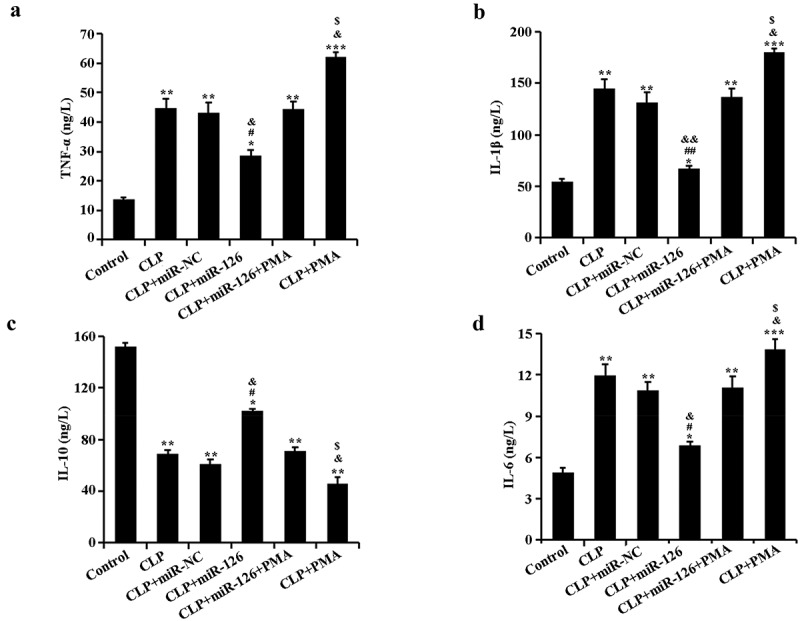
Levels of TNF-α, IL-6, IL-1β and IL-10 in the serum of rats in different treatment groups

ELISA results revealed that in comparison with the control group, TNF-α, IL-6, and IL-1β pro-inflammatory factor levels in rat serum in CLP group raised remarkably, while IL-10 anti-inflammatory factor level dropped. After activation of NF-κB signaling pathway in CLP rats (CLP+PMA group), rats exhibited higher levels of inflammatory factors. After overexpression of miR-126, serum expression of pro-inflammatory factors was decreased and expression of anti-inflammatory factors was increased in CLP rats. In contrast, the inhibitory effect of miR-126 on inflammatory factors in CLP rats was diminished after concomitant activation of NF-κB signaling pathway (Figure 4a-D, P < 0.05). The above data conclusively indicated that miR-126 could significantly decrease serum pro-inflammatory factors and raise anti-inflammatory factor levels in CLP rats, thereby alleviating septic rats’ inflammatory responses.

Serum was collected from each group of rats. A. Detection of TNF-α expression in rats of each group by ELISA; B. Detection of IL-1β expression in rats of each group by ELISA; C. Detection of IL-10 expression in rats of each group by ELISA; D. Expression of IL-6 in rats of each group was detected by ELISA. *P < 0.05, ** P < 0.01 and ***P < 0.001 vs. control group; ^#^ P < 0.05, ^##^P < 0.01 vs. CLP + miR-NC group; ^&^P < 0.05, ^&&^P < 0.01 vs. CLP + miR-126 + PMA group; ^$^P < 0.05 vs. CLP group.

### 2.5 Effects of miR-126 on CAT, SOD and MDA activities in brain tissue of septic rats

**Figure 5.**
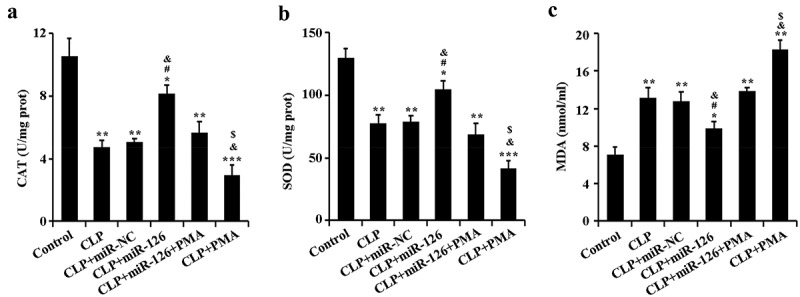
Activities of CAT, SOD and MDA in brain tissue of rats in different treatment groups

The effect of miR-126 on oxidative stress in septic rats was further determined. ELISA results showed that the activities of CAT and SOD were significantly lower and the activity of MDA was significantly higher in the brain tissues of CLP rats compared with the control group. The activities of CAT and SOD in the brain tissue of septic rats were significantly increased and the activities of MDA were significantly decreased after overexpression of miR-126. The levels of the above oxidative stress molecules were reversed upon overexpression of miR-126 along with activation of NF-κB signaling pathway (Figure 5a-C, P < 0.05). From the above results, it can be concluded that miR-126 was able to increase CAT and SOD activity and decrease MDA activity in the brain tissue of septic subjects.

A. Detection of CAT activity in rats of each group by ELISA; B. Detection of SOD activity in rats of each group by ELISA; C. The activity of MDA in rats of each group was detected by ELISA. *P < 0.05, **P < 0.01, ***P < 0.001 vs. control group; ^#^P < 0.05 vs. CLP + miR-NC group; ^&^P < 0.05 vs. CLP + miR-126 + PMA group; ^$^P < 0.05 vs. CLP group.

### 2.6 Effect of miR-126 on NF-κB signaling pathway

**Figure 6.**
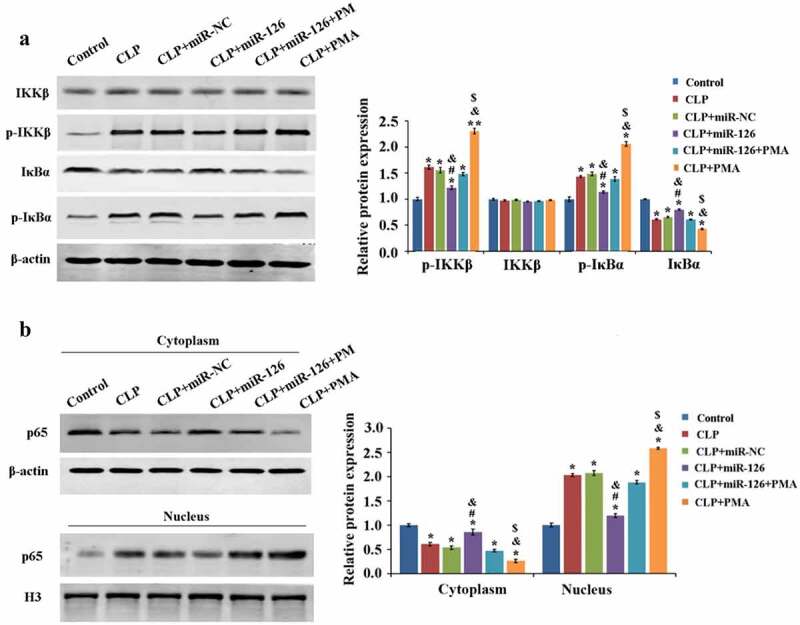
Effects of different treatments on blood-brain barrier related proteins and NF-κB signaling pathway in rat brain tissue

Finally, we analyzed the molecular mechanism of miR-126 inhibition of CLP-induced brain injury in septic rats. Compared with the control group, the protein expression levels of IκBα and p65 (cytoplasm) were significantly decreased and the expression levels of p-IKKβ, p-IκBα and p65 (nucleus) were significantly increased in the brain tissue of rats in the CLP group, which activated the NF-κB signaling pathway activity, while overexpression of miR-126 inhibited the NF-κB signaling pathway activity. Compared with the CLP+miR-126 group, the NF-κB signaling pathway activity was activated again in the CLP+miR-126+ PMA group (Figure 6a-C, P < 0.05). To conclude, miR-126 was able to inhibit NF-κB signaling pathway activity and to reduce NF-κB signaling pathway activation by PMA.

A. Western blot was used to detect the expressions of IKKβ, IκBα, p-IKKβ and p-IκBα in rats of each group; B. Western blot was used to detect the expression of p65 in the nucleus and cytoplasm of rat brain tissue in each group. *P < 0.05, **P < 0.01 vs. control group; ^#^P < 0.05 vs. CLP + miR-NC group; ^&^P < 0.05 vs. CLP + miR-126 + PMA group; ^$^P < 0.05 vs. CLP group.

## 3 Discussion

MiRNAs are ubiquitous in eukaryotes, and they are highly conserved, playing various functions in the development of individuals or diseases [19], such as promoting nerve development and cancer development. This study observed that the level of miR-126 expressed in SAE brain tissue reduced noticeably. Moreover, if miR-126 level climbed in CLP rats, it could greatly improve the neurobehavioral assessment scores in septic rats, indicating that miR-126 could avoid the damage of sepsis to the brain tissue of rats. Studies have shown that the damage of sepsis to brain tissue is mainly in the destruction of BBB, exposing brain tissue to the pathogenic environment and increasing the risk of brain injury [20]. Also, Pan et al. have described that the decrease of miR-126 expression is essential for the damage of BBB generated by arterial occlusion [21]. Fu et al. pointed out that increasing the expression of miR-126 can reduce the damage of BBB caused by cerebral hemorrhage [22]. In this study, increased miR-126 expression in CLP rats can noticeably lower the elevation of brain water content and EB extravasation brought on by sepsis and restore BBB-related proteins-claudin-5 and occludin expressions, indicating that upregulating the expression of miR-126 in CLP rats can reduce sepsis-induced BBB damage. In other words, the damage of sepsis to brain tissue might be caused by the downregulation of miR-126 expression.

Nowadays, studies believe that one of the mechanisms of sepsis-induced severe infection is causing an imbalance between inflammatory factors and anti-inflammatory factors [23]. Studies have pointed out that sepsis-induced CAT and SOD activity decreased significantly and MDA activity increased significantly. These changes contribute to one of the reasons for the destruction of the BBB, that is, reducing the protective effect of oxidative stress on nerves [24]. CAT and SOD have the effect of scavenging oxidative free radicals (ROS), and MDA is regularly used to evaluate peroxidation levels. In this study, pro-inflammatory cytokine (TNF-α, IL-6, and IL-1β) levels and serum MDA activity in CLP rats were remarkably suppressed by miR-126 expression, while anti-inflammatory cytokine (IL-10) levels and CAT and SOD activities elevated, thereby alleviating persistent damage caused by excessive inflammation and oxidation response.

Multiple studies have pointed out that NF-κB signaling pathway is the key to brain injury. Qu et al. pointed out that NF-κB signaling pathway activity inhibition can slow down the disturbance by subarachnoid hemorrhage to brain tissue [25]. Li et al. found that inhibiting the signaling pathway can avoid the damage caused by traumatic brain injury (TBI) to brain tissue [26]. The basis of inducing cerebral injury is NF-κB signaling pathway activation. Therefore, this study further explored the relationship between miR-126 and NF-κB signaling pathway using western blot. The data suggested that miR-126 could significantly reduce p-IKKβ and p-IκBα expressions and increase IKKβ expression, resulting in a reduction in the p-IKKβ/IKKβ and p-IκBα/IκBα ratios. Results have also shown that overexpressed miR-126 in CLP rats might inhibit NF-κB signaling pathway activity and interfere with PMA activation, thereby exerting the protective effect of miR-126 on brain tissue and alleviating SAE brain injury. According to other studies, unphosphorylated IKKβ and IκBα can keep NF-κB (p-65) in the cytoplasm, and prevent pMu65 from transferring to the nucleus and initiating stress responses such as apoptosis, causing damage to cells [27]. In contrast, p-IKKβ and p-IκBα would promote the nuclear transfer of p-65, causing damage to cells. In this study, by detecting p-65 in cytoplasm and nucleus, it was confirmed that miR-126 could inhibit the transfer of p-65 from the cytoplasm to the nucleus and avoid the damage of p-65 to cells.

## 4 Conclusions

In a nutshell, miR-126 expression was lowered in brain tissue of septic rats. Overexpression of miR-126 can improve brain injury in CLP rats through the inhibitions on NF-κB signaling pathway activity, inflammatory response and oxidation stress after brain injury. However, the molecular mechanism of miR-126 regulating NF-κB signaling pathway and its role in sepsis-induced brain injury remain to be further explored.

## Data Availability

Not Applicable

## Highlights

1. MiR-126 is down-regulated in the brain tissue of cecal ligation and perforation (CLP)-induced rats.
2. MiR-126 overexpression improves brain injury and BBB marker protein level in CLP rats.
3. MiR-126 overexpression inhibits inflammation and excessive oxidative stress in CLP rats.
4. MiR-126 overexpression suppresses NF-κB signalling pathway activity.
